# Proposing a re-conceptualisation of competency framework terminology for health: a scoping review

**DOI:** 10.1186/s12960-019-0443-8

**Published:** 2020-02-21

**Authors:** Jody-Anne Mills, James W. Middleton, Alison Schafer, Siobhan Fitzpatrick, Stephanie Short, Alarcos Cieza

**Affiliations:** 1 Department of Noncommunicable Diseases, World Health Organization, Geneva, Switzerland; 2 John Walsh Centre for Rehabilitation Research, Northern Clinical School, Faculty of Medicine and Health, University of Sydney, Sydney, Australia; 3 Department of Mental Health and Substance Use, World Health Organization, Geneva, Switzerland; 4 Health Workforce Department, World Health Organization, Geneva, Switzerland; 5 Faculty of Medicine and Health, University of Sydney, Sydney, Australia

**Keywords:** Competency framework, Competence, Conceptualisation

## Abstract

**Background:**

Competency frameworks are being taken up by a growing number of sectors and for a broad range of applications. However, the topic of competency frameworks is characterised by conceptual ambiguity, misunderstanding and debate. Lack of consistency in the conceptualisation and use of key terminology creates a barrier to research and development, consensus, communication and collaboration, limiting the potential that competency frameworks have to deal with real workforce challenges. This paper aims to advance the field by conducting a detailed review of the literature to understand the underlying causes of conceptual differences and divergent views and proposing a re-conceptualisation of competency framework terminology for use by the health sector.

**Methods:**

A broad scoping review of literature was conducted to identify publications relating to the conceptualisation of competency frameworks and key terms, examine how they are conceptualised and determine how this evolved. In addition, a purposive sample of health-related competency frameworks was chosen to illustrate how the terms and concepts are currently being applied in the health context.

**Results:**

Of the 4 155 records identified, 623 underwent text searches and broad quantitative analysis, and 70 were included for qualitative analysis. Quantitative analysis identified 26 key terms, which were coded under six thematic headings. Qualitative analysis using the thematic areas revealed two distinct conceptualisations of competency frameworks and their terminology emerging concurrently in the education and employment sectors, with different underpinnings and purposes. As competency frameworks have developed, these two conceptualisations intertwined, resulting in the same terms being used to convey different concepts. Examination of health-related frameworks showed that this merging of concepts is prominent, with lack of consistency in definitions and use of key terms even within a single organisation.

**Discussion and conclusions:**

Building on previous efforts to address the lack of conceptual clarity surrounding competency frameworks, this paper proposes a re-conceptualisation of the terminology that encompasses two distinct competency framework interpretations, using a glossary of mutually exclusive terms to differentiate concepts. The re-conceptualisation holds relevance for multiple competency framework applications within health, enabling harmonisation, clear communication, consensus-building and effective implementation of competency frameworks.

## Background

Competency frameworks have been widely used for several decades and are being taken up by a growing number of sectors seeking to articulate successful performance and its prerequisites. Educators, regulators, workforce strategists, evaluators and managers are among the many stakeholders establishing competency frameworks to build consensus, foster collaboration and promote standardisation [1–8]. While there appears to be reasonable agreement about what competency frameworks are as an organised collection of related competency statements, significant variation exists in how key terms are interpreted and applied, making useful comparisons among competency-based approaches difficult [9–12].

Critiques about the conceptual ambiguity of competency framework terminology are well documented, with the underlying concepts being described as “fuzzy” [13], “shifting sand” [3] and an uncertain foundation on which to build a framework [10, 11, 14]. The reason for this, while debatable, may result from the adoption of the terms in frameworks developed in different contexts and a lack of clarity around how they relate to different purposes [8, 15–18]. What is consistently acknowledged is the confusion that exists due to inconsistent interpretation and use of terms [14].

While the pitfalls of conceptual uncertainty are evident, there have been few notable attempts to establish a universal set of definitions within or between sectors [19–21]. Harmonisation and standardisation of definitions is clearly required if competency frameworks are to realise their potential as tools that can be applied in a wide range of circumstances. The overarching objective of this study is to fulfil this need by proposing a re-conceptualisation of competency framework terminology, supported by definitions that can be adopted in and beyond the health sector. To achieve this objective, this scoping review aimed to:

1. Identify key terms related to competency frameworks;
2. Determine how the conceptualisation of key terms evolved and how they are used; and
3. Explore how the key terms are defined and used in the context of health-related competency frameworks.

## Methods

### Scoping review

To capture the breadth of information necessary to meet the aims, a scoping review of literature from all sectors was conducted. The authors applied the methodological steps defined by Arksey and O’Malley to ensure a systematic and rigorous approach in developing the research questions, identifying publications, including and excluding publications, extracting data, and reporting findings [22]. The following exploratory questions were defined:

Exploratory question for aim 1:

- What are the key terms related to competency frameworks?

Exploratory questions for aim 2:

b) Where did the conceptualisation of key competency framework terminology evolve?
c) How are key competency framework terms applied?

Exploratory question for aim 3:

d) How are key competency framework terms defined and applied in the context of health?

**Fig. 1:**
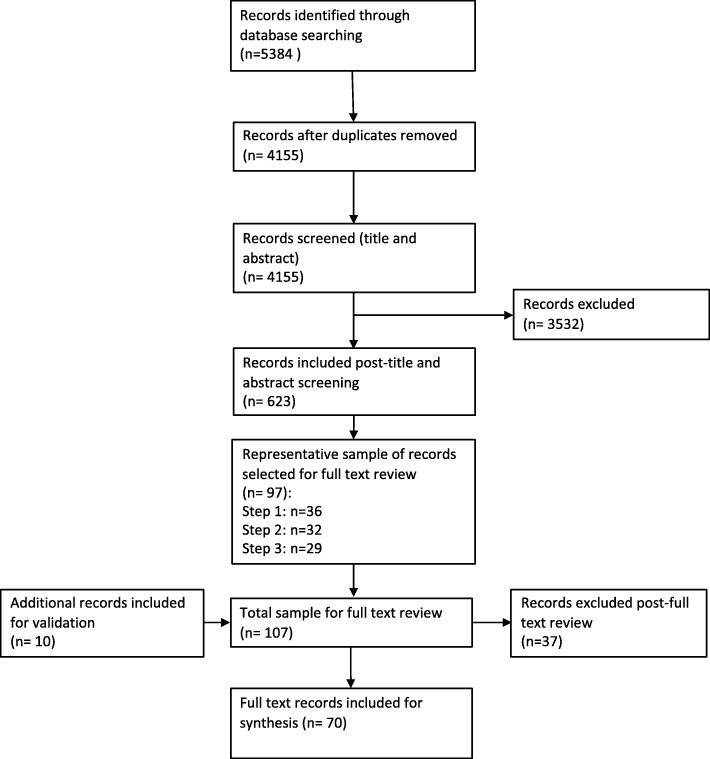
Scoping review results

Over the period between 6 and 13 August 2018, the first author (JM) searched Scopus and Web of Science using the broad terms “competenc* framework*” OR “competenc* model*”. Databases were selected to capture publications across different sectors so that the historical evolution of the competency framework terminology could be drawn from different fields, including education, industry, business and health. To capture the widest scope of literature, no filters were used for publication date, language or publication type. A modified PRISMA method was used to record the findings (see Fig. 1). Results were exported to Endnote, deduplicated using the Bramer method, and exported into the web application, Rayyan,^1^ with further duplicates identified and removed [23]. Titles and abstracts were screened by the first author according to their relevance to the exploratory research questions. Publications for which the term “competence” was used in a different context to that of the research questions, such as in a patient’s competence for decision making in a medical or legal context for example, were excluded, as were those focused solely on the technical content of a competency framework, without addressing the exploratory research questions. Publications with no abstract or for which no full text could be identified were also excluded. Where it was not possible to determine if a publication met the inclusion criteria, it was included for full text review.

### Data extraction and analysis

#### Aim 1: Identify key competency frameworks terms

The full texts from all of the records included post title and abstract screening (*n* = 623) were imported to NVivo 12 and a word frequency query run to identify key terms. Terms were thematically grouped and coded for further qualitative analysis. As new terms were identified through the full text review (*n* = 95, see details of three-stage process described below), additional text searches were run in NVivo to assess their frequency in the literature.

#### Aim 2: Capture the origin of dominant interpretations of key competency framework terminology, and how the terms are defined and used

Understanding where the key terminology was derived from and how these terms have been defined and used required full text review and data extraction. In order to make this feasible, a representative sample was selected using a three-stage process as detailed below:

1. Step 1 involved manually selecting all publications directly addressing the conceptualisation of competency frameworks, clarifying the precise meanings, interpretation and application of terms.
2. Step 2 included randomly selecting 5% of the remaining publications (using the randomisation function in Microsoft Excel), reflecting the same distribution of publication dates as the original included publications to ensure the historical evolution was captured. This percentage was considered by the authors to provide a quantity of publications that was feasible for full text review, considering the additional articles available from steps 1 and 3.
3. In step 3, the reference lists of the publications from steps 1 and 2 were screened for additional articles that directly addressed the exploratory research questions.

A REDCap^2^ database was created to extract information thoroughly and efficiently from the representative sample for each of the codes identified through NVivo. The first author undertook data extraction and synthesis, with findings validated through independent analysis of a smaller sample of publications by two additional authors (JWM and AS). The sample for each author included ten publications from step 1 (previously reviewed by the first author) and five new randomly selected publications from remaining included publications. On reviewing the full texts, the two authors extracted data into an Excel template based on the same codes used by the first author. Once completed, findings from the three authors were compared to identify discrepancies. The validation process did not identify any additional themes and confirmed the findings of the first author. Observations from the review of new publications were integrated into the data synthesis.

#### Aim 3: Explore how the key competency framework terms are defined and used in the context of health

Since many health-related competency frameworks exist, purposive sampling was used to select a number with which to examine how the key terms are defined and used. Identified competency frameworks published by the World Health Organization (WHO) were selected, as these covered diverse areas of health and enabled the authors to examine how terms were defined and used among different frameworks within a single organisation. Global or internationally recognised competency frameworks that appeared in the included literature were also selected. Definitions of the key terms were extracted, and competencies from the frameworks examined to observe how they had been applied.

## Results

Scoping of the electronic databases returned 4 155 results post de-duplication, of which 623 were deemed suitable for full text review (Fig. 1). After applying the three-staged method described above, a representative sample of 97 records were identified, and a further ten publications were included through the validation process (sample *n* = 107). Full text review against the inclusion and exclusion criteria resulted in a total of 70 records undergoing qualitative analysis (Table 1).

**Table 1:** Synopsis of included publications

	Number of publications	References
Sector		
Health	24	[1, 2, 9, 19, 24–43]
Other sector	34	[5, 6, 8, 10, 12, 13, 15–18, 44–67]
Cross-sectoral	12	[3, 4, 11, 14, 20, 68–74]
Publication year		
< 1990	1	[69]
1990–1994	6	[8, 34, 45, 52, 64, 68]
1995–1999	15	[9, 19, 20, 33, 38, 39, 46, 49, 57–59, 61, 65, 71, 73]
2000–2004	5	[2, 15, 16, 51, 55]
2005–2009	13	[3, 5, 10, 11, 14, 24, 28, 31, 32, 47, 50, 56, 60]
2010–2014	21	[1, 4, 6, 13, 17, 18, 26, 27, 30, 35–37, 40–42, 48, 54, 62, 63, 74]
2015–2019	9	[12, 25, 29, 43, 44, 53, 66, 67, 70, 72]
Country^a^		
Australia	3	[1, 9, 19
Belgium	1	[42]
Brazil	1	[44]
Canada	5	[10, 15, 27, 32, 43]
China	3	[13, 18, 63]
Croatia	1	[35]
Czech Republic	1	[54]
Denmark	1	[36]
Finland	1	[24]
France	2	[5, 14]
Germany	2	[47, 53]
Greece	2	[56, 62]
Iran	1	[37]
Italy	1	[25]
Lithuania	1	[6]
Malaysia	1	[12]
Morocco	1	[72]
Netherlands	2	[30, 70]
New Zealand	2	[3, 49]
Singapore	1	[55]
Tunisia	1	[74]
United Kingdom	19	[8, 26, 28, 29, 31, 33, 34, 39–41, 45, 51, 52, 59, 61, 64, 66, 68, 71]
United States of America	17	[2, 4, 11, 16, 17, 20, 38, 46, 48, 50, 57, 58, 60, 65, 67, 69, 73]

^a^Country where research was conducted or, if not relevant, country of first author’s affiliation

### Key competency framework terminology

**Fig. 2:**
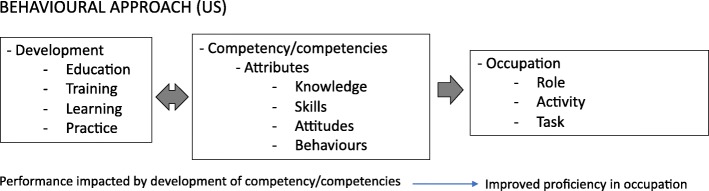
Relationship between key terms based on educators’ behavioural approach

Word frequency and text searches in NVivo and coding of full texts identified five competence/competency “like terms”, five “attribute^3^ terms”, ten “application” terms, three “development” terms, three “occupation” terms and three “related concepts” (see Table 2). This list is not exhaustive (additional attributes were described by authors); however, they represent the most frequently represented terms for each thematic area. Qualitative analysis of the full texts allowed the authors to examine how the “like terms” and “attributes” are conceptualised and how this relates to their use. These relationships are depicted in Fig. 2.

**Table 2:** Key competency framework terms identified in the literature

Code/thematic area	Term	Number of appearances in records (*n* = 623)
Like terms	Competency	24 769
	Competencies	21 167
	Competence	13 966
	Competences	3616
	Competent	1336
Attributes	Skill(s)	12 394
	Knowledge	9874
	Behaviour(ior)	6611
	Values	1963
	Attitudes	1170
Applications	Management	11 925
	Research	8919
	Assessment	6977
	Standard(s)(ize)(ise)	4334
	Communication	2812
	Recruit(ment)	766
	Regulate(ion)(s)	677
Development	Practice	8806
	Training	7840
	Learning	7176
Occupation	Activity(ies)	4045
	Role	3894
	Task(s)	3706
Relating concepts	Performance	8282
	Level	6974
	Proficiency(t)	440

### The evolution of the concepts underlying competency framework terminology

From the literature, it is apparent that two dominant conceptualisations of competency framework terminology emerged, almost concurrently, in the United States of America (US) and United Kingdom (UK) responding to different perceived needs and motivations within the education and employment sectors respectively [13, 17, 20, 46, 58]. The emergence of these conceptualisations is addressed in turn.

#### The emergence of behavioural competency frameworks by educators in the US in the 1970s

Competency frameworks came to prominence in the education sector when David McClelland, a well-known Harvard professor and psychologist, proposed in 1973 that competency was a superior indicator of occupational performance than traditionally used IQ tests [13, 17, 20, 58]. He described competency as: “generic bodies of knowledge, motives, traits, self-image and social roles and skills, that are causally related to superior or effective performance” [13] pp. 679. According to this definition, “competency” is underpinned by the concept of accumulated attributes and is linked to performance in general, rather than to a specific occupation or activity. Within the education sector, which is primary concerned with the students’ development, competency is also viewed as continuous and evolving [66]. Indeed, the term “competent”, which denotes a definitive state or end point, is to some degree incongruent with this view. This approach shaped the conceptualisation of competency framework terminology in the sector, notably associating competency with the development of attributes, which are applied in work (see Fig. 2). This behavioural conceptualisation and the relationship between terms that it represents are distinct from that which emerged in the UK from the employment sector.

#### The emergence of the functional competency frameworks in the UK by employers in the 1980s

**Fig. 3:**
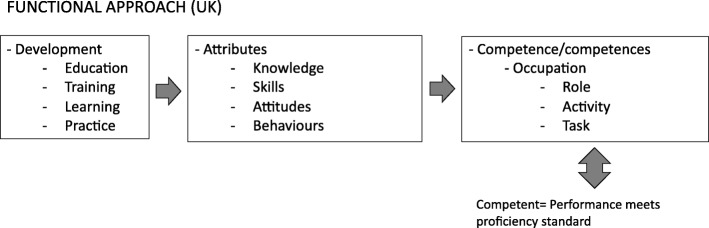
Relationship between key terms based on the employers’ functional approach

The rise of competency frameworks in the UK and the conceptualisation of their terminology were driven by the rise of worker rights and the growing demand for standardised selection criteria in recruitment. Employers in industry needed to develop standards for occupational performance based on expected outcomes, which were used to unify work-based qualifications [46]. Unlike the behavioural approach, competency frameworks in the UK were designed to capture the performance expectations for specific occupations [15, 26]. This interpretation was aptly termed the “functional-analysis approach” (hereon referred to as the “functional approach”) [15] pp. 9. Competency frameworks developed according to the functional approach were designed to reflect standards—a defined level of performance expected by an employer. The term “competence(s)” was therefore used to portray a dichotomous concept, whereby one either achieved the standard (was competent) or did not [59]. For example, in relation to recruitment and assessment in employment, Christopher Rowe stated that, “competence can only be measured on a pass/fail basis: people are either competent or they are not. This is determined by whether or not a person reaches a measurable standard…” [59] pp. 14. Furthermore, competence is defined solely within the context of occupational roles and are defined as activities or tasks. Unlike the behavioural approach, attributes are thus considered distinct from competence (see Fig. 3).

The disparity between the behavioural and functional approaches and the associated conceptual distinction between their terminology, summarised in Table 3, highlight that the two approaches were designed to achieve different ends by different groups. Thus, the terms can be used and interpreted in multiple ways, perpetuating ambiguity. Grzeda (2004), who straddles the two approaches in his role as a professor in the area of management, concluded that “resolving these competing [approaches] does not appear imminent and may in fact not be achievable since both appear to have some merit.” [10]

**Table 3:** The evolution and conceptualisation of terms based on the behavioural and functional approaches

Defining characteristics	Evolution and conceptualisation of terms based on the behavioural approach	Evolution and conceptualisation of terms based on the functional approach
Originating sector	Education	Employment
Originating country	US	UK
Application	Curriculum development, education and training	Employment, standardisation, and workforce regulation
Motivation	Supporting attainment of the highest level of proficiency	Achieve highest production at lowest cost
Focus	Development of competence (continuous)	State of competence (dichotomous)
Primary question	What does a person need to perform effectively? *Or* How does a person perform effectively?	What is effective performance?
Emphasis	Inputs	Outcome
Describes	Attributes of a person, i.e. knowledge, skills, attitudes and behaviours	Roles, activities or tasks
Example	Communicates effectively	Performs a risk assessment

#### The merging of behavioural and functional approaches

**Fig. 4:**
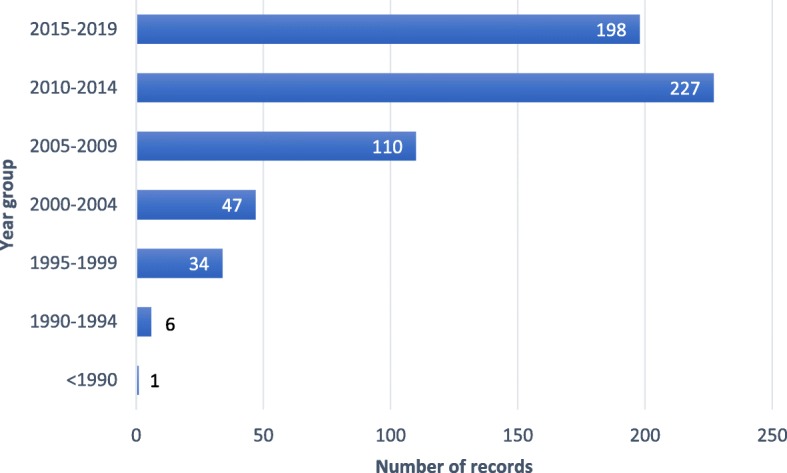
Distribution of records (*n* = 623) by year of publication over time, < 1990–2019. As no data extraction was required to establish the distribution of publications over time, it was feasible to include all 623 records that were included post title and abstract screening

Over time, competency frameworks have been taken up beyond the education and employment sectors, and outside the US and UK, where they came to prominence [11, 13, 14]. This evolution was characterised by a merging of the behavioural and functional approaches, as competency frameworks took on a wider range of applications associated with either or both of the education and labour sectors. The merging meant that competency frameworks used the same terms but often attributed different concepts to them, with variable cohesion observed between which concept was attributed and the motivation/application of the framework [15–18, 20]. Consequently, the proliferation of competency frameworks seen since 2005 (illustrated in Fig. 4) has been accompanied by growing confusion. This is potentially most evident in the degree of variation seen in how terms are used in competency frameworks.

### The use of terminology in competency frameworks

As the behavioural and functional approaches became intertwined, key terms were applied differently in competency frameworks. Frameworks apply the terms “competency(ies)” or “competence(s)” to either describe *how* performance is achieved (behavioural approach) or *what* performance looks like in the context of an occupation (functional approach), or a combination of both [31]. There is a tendency for the term “competency(ies)” being used to describe the *how* and “competence(s)” being used to describe the *what*, but this is largely inconsistent [1, 26, 31, 57, 61]. Furthermore, as competency frameworks are taken up beyond English-speaking countries, the nuance between these terms is lost. It is not surprising that criticisms have arisen since these like terms represent distinct concepts that serve different applications in different frameworks. For example, educationalists tend to criticise competency frameworks that use the term “competency” or “competence” to describe activities or tasks and the standards to which they should be conducted as reductionist, claiming that they fail to capture the complexities of practice and the range of attributes that underlie successful performance [1, 6, 9, 10, 32, 33, 43, 71]. They argue that it is not possible to define a profession or role by discrete tasks and that these are meaningless outside of their real-world application. Conversely, those from the industry point to the issues associated with using the term to describe personal attributes, such as empathy, imagination, reflection and resilience. They note the inferences needed for their assessment and claim that they erode the validity of competency frameworks as tools for performance measurement [1, 3, 11, 55].

There does not appear to be consistency in use of umbrella terms for knowledge, skills, attitudes, values and other attributes (attributes being used in this paper), and how they are defined was not a frequent theme in the literature. It was apparent, however, that there is widespread confusion surrounding their use, as this is inevitably tied to the conceptualisation of competency/competence on which a framework is built. When this is unclear, which it frequently is, further debate arises. Some frameworks focus solely on activities and tasks and do not explicitly include underlying knowledge, skill or other attributes (Frameworks 2–4 from Table 4 are examples) [76–78]. This was flagged in one publication as “a prime reason why so many people lapse into a narrow view of competency [frameworks]” [19], pp., 2. Adding to the confusion is the fact that attributes are commonly referred to as “competencies”. Early on, Woodruffe (1993) identified this issue and observed that “calling [attributes] ‘competences’ is likely only to muddle the definition of a competency again, and it seems better to use a separate label” [64]. He did not, however, offer such a label.

**Table 4:** Competency terminology in a sample of existing health-related competency frameworks

Framework		Definitions and examples	Interpretation reflected
1	Integrating HIV-related content into a competency-based curriculum. 1993 WHO, Regional Office for the Western Pacific [75] *http://www.who.int/iris/handle/10665/206922*	*Definition* “Competence”: Competence is the ability to effectively and efficiently deliver a specified professional service. This implies that the nurse is able to practise at a proficiency (mastery of learning) in accordance with local conditions to meet local needs.	Functional
		*Examples* • Describes universal precautions in infection control. • The student takes a sexual history of the HIV positive person or a person with AIDS.	Functional (and knowledge)
2	Sexual and reproductive health core competencies in primary care: attitudes, knowledge, ethics, human rights, leadership, management, teamwork, community work, education, counselling, clinical settings, service, provision. 2011 WHO [76] *https://apps.who.int/iris/bitstream/handle/10665/44507/9789241501002_eng.pdf?sequence=1&isAllowed=y*	*Definition* “Competence”: Sufficient knowledge, psychomotor, communication and decision-making skills and attitudes to enable the performance of actions and specific tasks to a defined level of proficiency.	Functional
		*Examples* • The primary health-care team member/s provide high-quality health education related to sexual and reproductive health and sexual and reproductive health services. • The primary health-care team member/s provide high-quality family-planning care	Functional
3	CanMEDS Terminology in Medical Education Project: Glossary of Terms. 2012 Royal College of Physicians [77] *http://www.royalcollege.ca/rcsite/canmeds/canmeds-framework-e*	*Definition* “Competence”: The array of abilities across multiple domains or aspects of physician performance in a certain context. Statements about competence require descriptive qualifiers to define the relevant abilities, context, and stage of training or practice. Competence is multi-dimensional and dynamic. It changes with time, experience, and setting. “Competency”: An observable ability of a health professional related to a specific activity that integrates knowledge, skills, values and attitudes. Since competencies are observable, they can be measured and assessed to ensure their acquisition. Competencies can be assembled like building blocks to facilitate progressive development	Behavioural
		*Examples* • Plan and perform procedures and therapies for the purpose of assessment and/or management • Demonstrate a commitment to high-quality care of their patients	Functional and behavioural
4	Pharmacy Education Taskforce: A Global Competency Framework. 2012 International Pharmaceutical Federation [78]*.* *https://**www.fip.org/files/fip/PharmacyEducation/GbCF_v1.pdf*	*Definition* “Competence”: Knowledge, skills, behaviours and attitudes that an individual accumulates, develops, and acquires through education, training, and work experience.	Behavioural
		*Examples* • Advise on health promotion, disease prevention and control, and healthy lifestyle • Tailor communications to patient needs	Functional and behavioural
5	Four-year, integrated nursing and midwifery competency-based, prototype curriculum for the African Region. 2013 WHO, Regional Office for Africa [79] *https://apps.who.int/iris/bitstream/handle/10665/254742/9789290232612eng.pdf?sequence=1&isAllowed=y*	*Definition* “Competency”: Basic knowledge, skills, attitudes and judgement required to safely perform the prescribed role.	Functional
		*Examples* • Interact effectively with clients fostering mutual respect and shared decision making to enhance client satisfaction and health outcomes. • Conducts a comprehensive assessment of a client in a caring, respectful and culturally sensitive manner.	Functional and behavioural
6	Core competencies in adolescent health and development for primary care providers including a tool to assess the adolescent health and development component in pre-service education of health-care providers. 2015 WHO [80]. *https://apps.who.int/iris/bitstream/handle/10665/148354/9789241508315_eng.pdf?sequence=1*	*Definition* “Competency”: Sufficient knowledge, psychomotor, communication and decision-making skills and attitudes to enable the performance of actions and specific tasks to a defined level of proficiency.	Functional
		*Examples* • Effectively interact with an adolescent client • Assess normal growth and pubertal development and manage disorders of growth and puberty	Functional and behavioural
7	WHO Competency Framework for Health Workers’ Education and Training on Antimicrobial Resistance. 2018 WHO [81]. *http://apps.who.int/medicinedocs/documents/s23443en/s23443en.pdf*	*Definition* “Competency”: A “combination of knowledge, skills, motives and personal traits”, development of which should help individuals to continually improve their performance and to work more effectively.	Behavioural
		*Examples* • Health worker understands and implements the principles of hygiene, sanitation and IPC to reduce the spread of AMR • Health worker demonstrates that they have the knowledge and understanding… to facilitate optimal and safe use of antimicrobial agents for management of infections.	Behavioural

### The conceptualisation of competency framework terminology in health

**Fig. 5:**
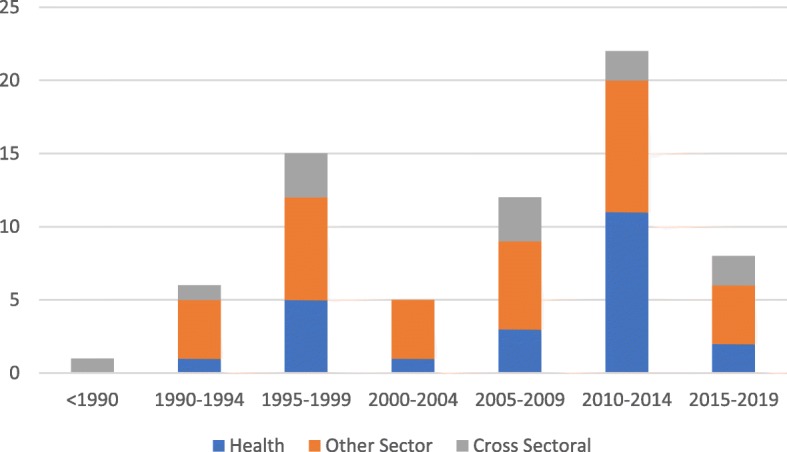
Distribution of records (*n* = 70) by sector over time, < 1990–2019

**Fig. 6:**
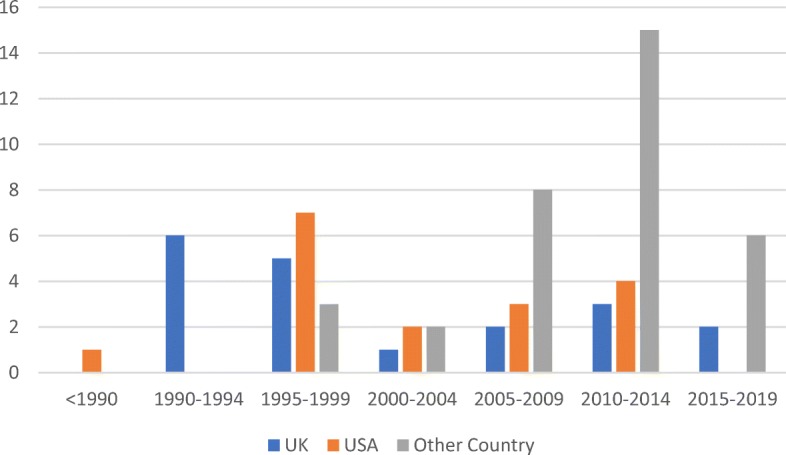
Distribution of records (*n* = 70) by country of publication over time, < 1990–2019

The third aim of this study was to examine how key terms are conceptualised and applied in the context of health. An examination of several health-related competency frameworks revealed a haphazard intermingling of the behavioural and functional approaches in definitions and use of terms, which is not unexpected given the inherent role that health plays in both education and training, as well as employment and performance management of workers (Table 4) [75–81]. Health-related competency frameworks represented 40% of the 70 records that underwent full text review, representing a considerable interest within the health sector. As seen in Fig. 5, publications of health-related competency framework literature peaked in the years 2010–2014 (46% of health-related records included in the review (*n* = 28) were published in this window), immediately following the 2010 Lancet Commission report into health professions education that called for competency-based education and training [82]. This growth also coincides with the increasing attention workforce received in the health sector over this period, which culminated in a World Health Assembly resolution (WHA67.24) on human resources for health, and the subsequent publication of the Global Strategy on Human Resources for Health: Workforce 2030 [83]. Interestingly, of the 70 included publications, 38% of those derived from countries other than the US or UK (Fig. 6) were from the health sector (as opposed to all other sectors and cross-sectoral studies). The expansion of competency frameworks beyond the countries from which the behavioural and functional conceptualisations derived may further explain the conflation of definitions and uses of terminology observed in health-related competency frameworks.

Table 4 presents the definitions of competency (or like terms, as available) of health-related competency frameworks (presented in chronological order) and extracts examples of their use in competency statements. These are then mapped to the approach that they reflect (behavioural, functional or hybrid of both). It is apparent that definitions are variable, as are their use, and that there is often an incongruence in approaches between and within frameworks. Four frameworks in the sample use definitions aligned with a functional approach (frameworks 1, 2, 5 and 6), articulating a specific level of proficiency and the ability to perform a specific role, action or task. Three definitions aligned with a behavioural approach (frameworks 3, 4 and 7), explicitly highlighting the development of proficiency without making reference to any specific role, activity or task.

The presence of both functional and behavioural approaches in the definitions of competence/competency appears even within a single organisation; four definitions from WHO aligned with the functional approach and one with the behavioural. The conflation is most prominent, however, in the application of the terms; four of the seven frameworks included competencies that aligned with both approaches irrespective of the conceptual definition used. For example, framework 3 includes task-based competencies, such as planning and performing procedures (functional approach), and person-centric competencies, such as demonstrating commitment to high-quality care (behavioural). There is clearly a disparity between how the terms “competence” or “competency” are being conceptualised and how they are being used. There is also a disjointedness between the concept applied to the terms and the intended application of the framework. According to its title, framework 1 is intended to inform curriculum development—an education-related application that would theoretically align with a behavioural approach. However, in its definition of competence, reference is made to a specific level of proficiency, which is a feature associated with a dichotomous conceptualisation of competence characteristic of a functional approach. Specifications of an expected level of proficiency portray a standard by which one can be measured against and are associated with employment-related applications, such as regulation.

Examination of the definitions further revealed the integration of “personal” attributes within definitions aligned with the functional approach. Framework 4, for example, includes attitudes and judgement in its definition, which are not characteristic of a functional approach as their assessment requires higher levels of inference. Attributes, notably knowledge, also appeared to be conceptualised as competencies. This is seen in framework 1, where description of a specific area of knowledge was included as a competency. This reflects the concept of competency as attributes, rather than as the expression of their aggregation in behaviour.

## Discussion

Competency frameworks are becoming more widely used across a variety of sectors and for a broad range of applications. However, conceptual ambiguity and lack of consistency in the use of key terminology still vexes the field. This limits common understanding, cohesiveness and standardisation for research, development and implementation into practice. This scoping review has undertaken a systematic and comprehensive approach to identifying the underlying causes of the conceptual differences and diverging views and seeks to examine how this has influenced competency frameworks in the health sector. Two highly influential conceptualisations have emerged from the education and employment sectors, which have defined this topic area with seemingly incongruent views to competency. They consider competency as either “behavioural” (continuous and evolving, underpinned by the accumulation of attributes and linked to performance in general) or “functional” (related to the performance of a specific occupation or activity and concerned with a definitive end point of being “competent”).

The intertwining of the behavioural and functional approaches seen in the health-related competency frameworks presented in Table 4 may reflect the interconnectedness between the education and employment sectors. Indeed, education-related applications of frameworks (e.g. developing curriculum) are influenced by employment-related applications (regulation of proficiency) and vice versa. It appears evident that the developers of competency frameworks see the need for either or both the behavioural and functional approach, depending on the point of view of the developer and the frameworks’ intended application. Acknowledging this need and building on the findings of the scoping review, this study proposes a re-conceptualisation of competency frameworks, supported by a glossary of key terms (Table 5). This has been developed through a health lens, although it may be equally applicable to other areas. The glossary encompasses concepts from both the behavioural and functional interpretations but distinguishes between them through assigning discrete terms to each.

**Table 5:** Proposed glossary of terms for health-related competency frameworks

Term	Definition	Conceptual characteristics
Activity	An area of work that encompasses groups of related tasks. Activities are time limited, trainable and, through the performance of tasks, measurable.	Time limited, i.e. begins and ends Describe what is done
Attitude	A person’s feelings, values and beliefs, which influence their behaviour and performance of tasks.	An unobservable attribute inferred through performance
Behaviour	Observable conduct towards other people or activities that expresses a competency. Behaviours are durable, trainable and measurable.	Observable attribute, often applied in combination, i.e. several behaviours may contribute towards one competency
Competency	The observable ability of a person, integrating knowledge, skills, and attitudes in their performance of tasks. Competencies are durable, trainable and, through the expression of behaviours, measurable.	Not time limited, i.e. durable through multiple activities Can develop/improve or erode over time
Competent	Performance of required competencies and activities to a defined standard for an occupational role (e.g. “she/he is competent”).	Dichotomous, i.e. one is or is not competent
Knowledge	The informational base of competencies and activities.	An unobservable attribute of competence inferred through performance or determined through specific testing A competency and/or activity may draw on multiple areas of knowledge simultaneously
Proficiency	A person’s level of performance (e.g. novice or expert).	A degree of ability to perform (continuous)
Occupational role	A category that characterises certain groups of activities (e.g. student, practitioner, educator, manager, researcher).	An aggregate of linked activities that serve a common purpose The macro level to activities (meso) and tasks (micro) Determines scope of practice
Skill	A specific cognitive or motor ability that is typically developed through training and practice.	Observable (physical) and unobservable (cognitive) attribute, often applied in combination, i.e. several skills may contribute towards one competency and/or activity
Standard	The level of proficiency required to perform an occupational role, acquire a professional title, or be deemed safe to perform specific tasks.	A specific level of performance (discrete)
Task	Observable units of work as part of an activity, which draw on knowledge, skills, attitudes and behaviours. Tasks are time limited, trainable and measurable.	Observable attribute of activities, often applied in combination, i.e. several tasks may contribute towards one activity

### Proposed re-conceptualisation of competency framework terminology for health

**Fig. 7:**
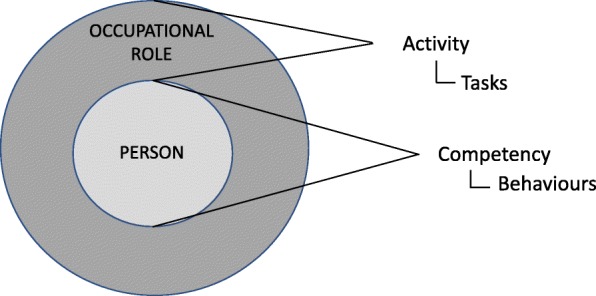
Differentiating between competency as person-centric and activity as role-centric

**Fig. 8:**
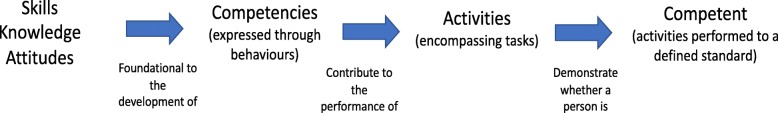
Relationship between key competency framework terminology according to proposed conceptualisation

Four features characterise the proposed re-conceptualisation of key terms:

1. Differentiation between “competency” and “activity” (see Fig. 7)
  The first distinction is necessary because competencies, being embodied by a person, translate across multiple roles, activities and tasks. How these competencies are expressed may differ depending on how they are contextualised. Activities can be differentiated from competencies in that they are time limited (they begin and end), while competencies are durable.^4^ For example, “communicates effectively” would be considered a competency, while, “conducts an intervention”, would be considered an activity.
  Both activities and competencies can be broken down into smaller component parts, enabling a more accurate and granular description. In this re-conceptualisation, it is proposed that tasks are the component parts of activities (given that an activity can encompass multiple tasks), and behaviours are the component parts of competency (in that competencies can be expressed through numerous behaviours). Using the examples provided above, a behaviour for effective communication may include using and interpreting body language, and a task associated with conducting an intervention may include prescribing an exercise programme. Notably, both tasks and behaviours are observable, an important factor for applications relating to practical assessment.

2. Distinguishing attributes from competencies and activities (Fig. 8).

Disentangling attributes (knowledge, skills, and attitudes) from competencies and activities enables greater standardisation of how they are expressed and organised in competency frameworks. Much of the current confusion and debate surrounding competency frameworks has resulted from a failure to recognise their conceptual distinction and doing so will go far to supporting greater comparability between frameworks.

Building on the previous examples, knowledge of cultural communication practices would underpin effective communication, and knowledge of the indications and contraindications of specific exercises would be required to prescribe an exercise programme. Skills and attitudes would similarly provide the necessary underpinnings for the performance of a competency or activity.

3. Recognising competency as evolving through increasing levels of proficiency

The concept of proficiency is a necessity for many applications of competency frameworks. The glossary articulates the term “standard” as describing the defined point of proficiency at which someone may be deemed “competent” in a specific context. For example, it would require a relatively low level of proficiency to prescribe a protocol-based exercise programme, as opposed to a customised programme that caters for individual factors. A competency framework may describe these different levels, but a standard would articulate at which a person would be considered “competent”.

4. Conduciveness to translation

Finally, the terms do not rely on fine nuances of the English language to differentiate them, and use plain-language, succinct definitions. For example, the term “competences”, which could not be differentiated from “competencies” in many languages, is not included. This is critical for the development and application of global competency frameworks in particular and for ensuring standardisation and effective communication between countries, cultures and groups.

While the glossary has been developed with the health sector in mind, it has the potential to be adopted by other sectors. At the time of publication, the concepts and terminology proposed here were applied to the WHO Rehabilitation Competency Framework (pending publication), which served as a test case for its application. The differentiation of concepts through distinct terms and the inclusion of both behavioural and functional interpretations has proved invaluable in organising information and providing clarity in this process.

### Existing contributions to competency framework terminology conceptualisation

The proposed re-conceptualisation builds on the reflections of several authors who have previously sought to synergise the behavioural and functional approaches. For example, in 1996, Paul Hager and Andrew Gonczi, two educationalists in Australia, presented a definition of competence intended to convey an “integrated approach” and a “richer conception of competence” [19]. The authors suggested that integrating attributes and tasks would enable competency standards to capture “the holistic richness of professional practice in a way that neither [the behavioural and functional] approaches could” (pp.15). More recently, Olle ten Cate proposed the concept of “Entrustable Professional Activities” (EPAs) in an effort to better connect competency frameworks to the workplace (creating a conceptual bridge between the education and employment sectors) [84]. EPAs are defined as a “units of professional practice, defined as tasks or responsibilities to be entrusted to the unsupervised execution by a trainee once he or she has attained sufficient specific competence” (pp. 157). Hager and Gonczi and ten Cate’s propositions are similar in so far as they both suggest that competence is underscored by a concept of integrated attributes and tasks. They differ, however, in that Hager and Gonczi posit that tasks should be described in general terms only with the emphasis being on the capabilities that underlie their successful performance, whereas ten Cate focuses attention on discrete tasks that are observable and measurable, leaving the underlying behavioural competencies to inference [85].

Others have also attempted to develop an operational definition that resolves the seemingly conflicting behavioural and functional approaches **[**3**,** 10**,** 11**,** 13**,** 38**,** 46**,** 54**,** 57**,** 59**,** 60**]**. For example, Woodruffe (1993), coming from an employment perspective, defined competency as “a set of behavioural patterns that the incumbent needs to bring to a position in order to perform its tasks and functions with competence” **[**64**]**. The concept of competency proposed by Woodruffe appears to focus on behaviours that can be linked to tasks, rather than an integration of these behaviours and the tasks that they are linked to, as proposed by Hager and Gonczi and ten Cate [19, 84]. The nuances between the authors’ conceptualisations of terms are subtle but not insignificant. Without consensus on whether the term “competency” encompasses tasks or links to them, confusion is bound to persist and comparability between frameworks will continue to be problematic. For this reason, the distinction between competencies and activities in the proposed re-conceptualisation of terms is considered critical. How a competency framework developer chooses to link competencies and activities may vary; they could be specifically mapped to each other, grouped under thematic domains or kept completely separate, depending on the preferred framework structure. Similarly, competency framework developers may choose to describe the behaviours through which competencies are expressed, and the tasks encompassing activities, or describe only the competencies and activities generally, depending on the level of specificity the intended application requires. The same holds true for the inclusion of attributes (knowledge, skills and attitudes), which may or may not be included according to their perceived value to the user. Regardless of competency framework arrangement or granularity, a common conceptual underpinning will certainly enhance comparability and bring clarity to future discourse [85].

## Limitations

The conclusions of this scoping review should be considered in light of its methodological limitations. Firstly, the sampling process applied to achieve feasibility for full text review meant that a large proportion of the literature that was potentially relevant (based on title and abstract screening) was not analysed (although all 623 records included post title and abstract screening were text-searched as per Table 2). While analysis of the 70 publications that did undergo full text review reached a saturation in themes, it is possible that additional themes or historical perspectives were missed. Secondly, data extraction and review for the majority of the publications was undertaken by one author. The validation process, whereby two additional authors conducted data extraction and review of samples of new and duplicate publications, sought to mitigate the risks associated with single-author data extraction and review. Nevertheless, multiple-author data extraction and review would have constituted a more robust methodology.

## Conclusions

The literature shows a trend of increasing interest in competency frameworks. Their relevance to numerous and increasing workforce challenges suggests that they will continue to be developed and used in the years to come. The conceptual ambiguity and subsequent debates and confusion that have plagued competency frameworks and eroded their credibility are driven by a conflation of two distinct conceptualisations of shared key terms. The re-conceptualisation presented here, and the glossary through which it is expressed, serves to bring clarity and insight in the future development and use of competency frameworks within health workforce education, recruitment, employment and regulation into the future.

## Data Availability

Raw data from the scoping review is available on request. Contact james.middleton@sydney.edu.au.

## Abbreviations

EPA: Entrustable Professional Activity
UK: United Kingdom
US: United States of America
WHO: World Health Organization

## Footnotes

1 https://rayyan.qcri.org/welcome

2 https://www.project-redcap.org/

3 In the absence of a consistent label, “attributes” is used in this paper to describe the (non-environmental) factors that underlie competency.

4 “Durable” refers to the ongoing presence of an ability, recognising that performance of the ability may not remain consistent

## References

[1] Thistlethwaite JE, Forman D, Matthews LR, Rogers GD, Steketee C, Yassine T (2014). Competencies and frameworks in interprofessional education: a comparative analysis. Acad Med.

[2] Calhoun JG, Davidson PL, Sinioris ME, Vincent ET, Griffith JR (2002). Toward an understanding of competency identification and assessment in health care management. Qual Manag Health Care.

[3] Markus L, Thomas HC, Allpress K (2005). Confounded by competencies? An evaluation of the evolution and use of competency models. New Zealand J Psychol.

[4] Campion MA, Fink AA, Ruggeberg BJ, Carr L, Phillips GM, Odman RB (2011). Doing competencies well: best practices in competency modeling. Pers Psychol.

[5] Belkadi F, Bonjour E, Dulmet M (2007). Competency characterisation by means of work situation modelling. Comput Ind.

[6] Sudnickas T, Kratavičiute-Ališauskiene A (2011). Analysis of applying competency models: case of the office of the prime minister of Lithuania. Public Policy Adm.

[7] Sherman RO, Bishop M, Eggenberger T, Karden R (2007). Development of a leadership competency model. J Nurs Adm.

[8] Munro A, ANdrews B (1994). Competences: dialogue without a plot? Providing context through business diagnostics. Exec Dev.

[9] Carberry C (1998). Contesting competency: cultural safety in advanced nursing practice. Collegian..

[10] Grzeda MA (2005). In competence we trust? Addressing conceptual ambiguity. J Manage Dev.

[11] Kuchinke KP, Hee-Young H (2005). Should caring be viewed as a competence? (re-) opening the dialogue over the limitations of competency frameworks in HRD. Hum Resour Dev Int.

[12] Ahmad J, Daud N, Pirzada K, Wickramasinghe D, Moens GA, Ahmed K (2016). Determining innovative tourism event professional competency for conventions and exhibitions industry: a preliminary study. Procedia Soc Behav Sci.

[13] Chen HM, Chang WY (2010). The essence of the competence concept: adopting an organization's sustained competitive advantage viewpoint. J Manag Organ.

[14] Le Diest FD, Winterton J (2005). What is competence?. Hum Resour Dev Int.

[15] Boritz JE, Carnaghan CA (2003). Competency-based education and assessment for the accounting profession: a critical review. Can Account Persp.

[16] Shippmann JS, Ash RA, Carr L, Hesketh B, Pearlman K, Batitsta M, Eyde LD, Kehoe J, Prien EP, Sanchez JI (2000). The practice of competency modeling. Pers Psychol.

[17] Lambert B, Plank RE, Reid DA, Fleming D (2014). A competency model for entry level business-to-business services salespeople. Serv Mark Q.

[18] Wu CH, Fang WC (2011). Combining the fuzzy analytic hierarchy process and the fuzzy Delphi method for developing critical competences of electronic commerce professional managers. Qual Quant.

[19] Hager P, Gonczi A (1996). What is competence?. Med Teach.

[20] Russ-Eft D (1995). Defining competencies: a critique. Hum Resour Dev Q.

[21] Ten Cate O. Medical education, competency-based. In: Cockerham WC, Dingwall R, Quah SR, editors. The Wiley Blackwell Encyclopedia of Health, Illness, Behavior, and Society. 1st ed: Wiley; 2014. https://onlinelibrary.wiley.com/doi/10.1002/9781118410868.wbehibs331.

[22] Arksey H, O’Malley LBC (2005). Scoping studies: towards a methodological framework. Int J Soc Res Methodol.

[23] Wichor M, Bramer DG, Gerdien B, de Jonge LH, Bekhuis T (2016). De-duplication of database search results for systematic reviews in endnote. J Med Libr Assoc.

[24] Ääri RL, Tarja S, Helena LK (2008). Competence in intensive and critical care nursing: a literature review. Intensive Crit Care Nurs.

[25] Bertoncello C, Buja A, Silenzi A, Specchia ML, Franchino G, Lazzari A (2015). Good governance competencies in public health to train public health physicians. Int J Public Health.

[26] Bruno A, Bates I, Brock T, Andersonm C (2010). Towards a global competency framework. Am J Pharm Educ.

[27] Chen SP, Krupa T, Lysaght R, McCay E, Piat M (2013). The development of recovery competencies for in-patient mental health providers working with people with serious mental illness. Adm Policy Ment Health Ment Health Serv Res.

[28] Clements R, Mackenzie R (2005). Competence in prehospital care: evolving concepts. Emerg Med J.

[29] Connolly M, Ryan K, Charnley K (2016). Developing a palliative care competence framework for health and social care professionals: the experience in the Republic of Ireland. BMJ Support Palliat Care.

[30] Czabanowska K, Burazeri G, Klemenc-Ketis Z, Kijowska V, Tomasik T, Brand H (2012). Quality improvement competency gaps in primary care in Albanian, Polish and Slovenian contexts: a study protocol. Acta Inform Med.

[31] Davis R, Turner E, Hicks D, Tipson M (2008). Developing an integrated career and competency framework for diabetes nursing. J Clin Nurs.

[32] Evans RJD, G. W. (2006). A model to describe the relationship between knowledge, skill, and judgment in nursing practice. Nurs Forum.

[33] Grant J (1999). The incapacitating effects of competence: a critique. Adv Health Sci Edu Theory Pract.

[34] Jinks AM (1994). Conceptualization of differing levels of educational attainment: what are the characteristics of nurses and midwives who have undertaken diploma and degree educational programmes?. J Nurs Manag.

[35] Meštrović A, Staničić Z, Hadžiabdić MO, Mucalo I, Bates I, Duggan C (2012). Individualized education and competency development of Croatian community pharmacists using the general level framework. Am J Pharm Educ.

[36] Morke AM, Dornan T, Eika B (2013). A response to “competency frameworks: universal or local” by Mortaz Hejri and Jalili (2012). Adv Health Sci Educ.

[37] Mortaz Hejri S, Jalili M (2013). Competency frameworks: universal or local. Adv Health Sci Educ.

[38] Nagelsmith L (1995). Competence: an evolving concept. J Contin Educ Nurs.

[39] Rolfe G (1997). Beyond expertise: theory, practice and the reflexive practitioner. J Clin Nurs.

[40] Smith L, Hawkins J, McCrum A (2011). Development and validation of a child health workforce competence framework. Commun Pract.

[41] Tsaroucha A, Benbow SM, Kingston P, Le Mesurier N (2013). Dementia skills for all: A core competency framework for the workforce in the United Kingdom. Demen-Int J Soc Res Pract.

[42] Vandeweerd JM, Cambier C, Romainville M, Perrenoud P, Desbrosse F, Dugdale A (2014). Competency frameworks: which format for which target?. J Vet Med Educ.

[43] Whitehead CR, Kuper A, Hodges B, Ellaway R (2015). Conceptual and practical challenges in the assessment of physician competencies. Med Teach.

[44] Barbosa JLV, Kich MR, Barbosa DNF, Klein AZ, Rigo SJ (2015). DeCom: a model for context-aware competence management. Comput Ind.

[45] Brown RB (1993). Meta-competence: a recipe for reframing the competence debate. Pers Rev.

[46] Dalton M (1997). Are competency models a waste?. Train Dev.

[47] De Coi JL, Herder E, Koesling A, Lofi C, Olmedilla D, Papapetrou O (2007). A model for competence gap analysis.

[48] Eagan P, Gutafson M, Vieth C (2012). Building a competency model for sustainability.

[49] Gray L (1999). New Zealand HRD practitioner competencies: application of the ASTD competency model. Int J Hum Resour Manage.

[50] Hayton JCM, G. M. (2006). Competencies in practice: an interview with Hanneke C. Frese Hum Resour Manage.

[51] Hood C, Lodge M (2004). Competency, bureaucracy, and public management reform: a comparative analysis. Governance..

[52] Iles PA (1993). Achieving strategic coherence in HRD through competence-based management and organization development. Pers Rev.

[53] Klotz VK, Winther E, Festner D (2015). Modeling the development of vocational competence: a psychometric model for economic domains. Vocat Learn.

[54] Kolibácová G (2014). The relationship between competency and performance. Acta Univ Agric Silviculturae Mendelianae Brun.

[55] Lievens F, Sanchez JI, De Corte W (2004). Easing the inferential leap in competency modeling: the effects of task-related information and subject matter expertise. Pers Psychol.

[56] Macris A, Papadimitriou E, Vassilacopoulos G (2008). An ontology-based competency model for workflow activity assignment policies. J Knowl Manage.

[57] McLagen PA (1997). Competencies: moving into the next generation. Train Dev.

[58] Mirabile RJ (1997). Everything you wanted to know about competency modeling. Train Dev.

[59] Rowe C (1995). Clarifying the use of competence and competency models in recruitment, assessment and staff development. Ind Commer Train.

[60] Sanchez JIL, E. L. (2009). What is (or should be) the difference between competency modeling and traditional job analysis?. Hum Resour Manage Rev.

[61] Stuart R, Thompson JE, Harrison J (1995). Translation. From generalizable to organization-specific competence frameworks. J Manage Dev.

[62] Thanopoulos C, Manouselis N, Stracke CM (2011). Understanding and adaptation of the concept of competences in the water sector. Agris On-line Pap Econ Inf.

[63] Wang L, James KT, Denyer D, Bailey C (2014). Western views and Chinese whispers: re-thinking global leadership competency in multi-national corporations. Leadership..

[64] Woodruffe C (1993). What is meant by a competency?. Leadersh Organ Dev J.

[65] Zingheim PK, Ledford GE, Schuster JR (1996). Competencies and competency models: does one size fit all?. ACA J.

[66] Bachkirova TS, C. L. (2015). From competencies to capabilities in the assessment and accreditation of coaches. Int J Evid Based Coach Mentor.

[67] Drisko JW (2015). Holistic competence and its assessment. Smith Coll Stud Soc Work.

[68] Burgoyne J (1993). The competence movement: issues, stakeholders and prospects. Pers Rev.

[69] Coit BF (1978). The concept of competence: an operational definition. Educ Technol.

[70] Fuller M, Heijne-Penninga M, Kamans E, van Vuuren M, de Jong M, Wolfensberger M (2018). Identifying competence characteristics for excellent communication professionals: A work field perspective. J Comm Manage.

[71] Hyland T (2003). Reconsidering competence. J Philos Educ.

[72] Idrissi MK, Hnida M, Bennani S (2016). Competency-based assessment: from conceptual model to operational tool.

[73] Idrissi MK, Hnida M, Bennani S. Competency-based assessment: from conceptual model to operational tool. In: Cano E, Iom G, editors. Innovative Practices for Higher Education Assessment and Measurement. 1st ed. Hershey: IGI Global; 2016. p. 57-78.

[74] Rezgui K, Mhiri H, Ghédira K (2012). Competency models: a review of initiatives.

[75] World Health Organization Regional Office of the Western Pacific (1993). Integrating HIV-related content into a competency-based curriculum.

[76] World Health Organization (2011). Sexual and reproductive health core competencies in primary care: attitudes, knowledge, ethics, human rights, leadership, management, teamwork, community work, education, counselling, clinical settings, service, provision.

[77] Royal College of Physicians and Surgeons of Canada (2012). CanMEDS: Better standards, better physicians, better care.

[78] International Pharmaceutical Federation (2012). Pharmacy Education Taskforce: A Global Competency Framework.

[79] World Health Organization Regional Office for Africa (2013). Four-year, integrated nursing and midwifery competency-based, prototype curriculum for the African Region.

[80] World Health Organization (2015). Core competencies in adolescent health and development for primary care providers including a tool to assess the adolescent health and development component in pre-service education of health-care providers.

[81] World Health Organization (2018). WHO Competency Framework for Health Workers’ Education and Training on Antimicrobial Resistance.

[82] Frenk J, Chen L, Bhutta ZA, Cohen J, Crisp N, Evans T, Fineberg H, Garcia P, Ke Y, Kelley P, Kistnasamy B, Meleis A, Naylor D, Pablos-Mendez A, Reddy S, Scrimshaw S, Sepulveda J, Serwadda D, Zurayk H (2010). Health professionals for a new century: transforming education to strengthen health systems in an interdependent world. Lancet.

[83] World Health Organization (2016). Global strategy on human resources for health: workforce 2030.

[84] ten Cate Olle (2005). Entrustability of professional activities and competency-based training. Medical Education.

[85] Ten Cate O (2013). Nuts and bolts of entrustable professional activities. J Grad Med Educ.

